# 2009 International Meeting of the International Psychogeriatric Association Third Congress of the Brazilian Association of Geriatric Neuropsychiatry

**Published:** 2008

**Authors:** João Carlos Barbosa Machado, Jerson Laks

This educative scientific meeting will take place in Rio de Janeiro from 4–7 May 2009 under the theme: Brain Aging and Quality of Life. We are convening an internationally renowned group of scientists who are experts in the field of research and clinical practice related to neurology, psychiatry and geriatric medicine to address issues in aging, neuroscience and mental health in old age. The International Meeting will provide a forum to meet and network with other clinicians, educators and researchers from all over the world.

We are looking forward to your participation. For more information, please visit http://www.abnpg.com/contatos.htm, call 55 31 3201 7905 or send an e-mail to exsecretaria@abnpg.com

**João Carlos Barbosa Machado****Jerson Laks**

